# Understanding collaborative consumption in the technology-driven sharing economy: A cost-benefit analysis perspective

**DOI:** 10.1371/journal.pone.0309024

**Published:** 2024-12-04

**Authors:** Ke Sun

**Affiliations:** Newcastle Business School, Northumbria University, Newcastle upon Tyne, United Kingdom; Critical Centrality Institute, MEXICO

## Abstract

Driven by the extensive implementation of information communication technology, collaborative consumption has become more popular. Historically, people have always thought that the best way to get something is to obtain the ownership of it. However, collaborative consumption has recently seen a meteoric rise in popularity due to that obtaining the right to use rather than own. More research into this emerging phenomenon is necessary, notwithstanding the huge impact that collaborative consumption activities have had on companies and individuals. Existing research indicates a lack of knowledge on the factors that motivate or impede user engagement in collaborative consumption. Building on the cost and benefit framework, this research presents a model that examines the effects of perceived benefits (enjoyment and economic reward), perceived costs (privacy risk and security risk) and perceived platform quality (system quality, service quality and information quality) on the intention to engage in the collaborative economy. Using a structural equation modelling approach, 524 active users with experience in car sharing evaluated the research model.The results show that perceived benefits and platform quality positively influence CC participation, the perceived cost reveals a partial support relationship to participate in CC, where security risks are supported but privacy risks are not. This research results will contribute to the research and practice on sharing economy.

## 1. Introduction

Driven by state-of-the-art technology and the widespread use of social media, the sharing economy has emerged as a powerful force for both innovation and economic influence. Many notable firms have used social media and advanced technology to create platforms and applications focused on shared consumption. The concept of shared consumption is centred on maximising the value of assets not completely used by their owners [1, 2]. In addition to meeting the diverse needs of customers, these platforms increase the worth of surplus personal possessions. Companies that adopt this cutting-edge business model for shared consumption effectively do not own anything themselves. Instead, they create networks that connect sellers and customers, making it easier to acquire products and services whenever needed, surpassing existing levels of utilisation. Uber and Airbnb, two rapidly growing multinational companies, have demonstrated their ability to create innovative business models that provide significant economic prospects [3, 4].

As services based on collaborative consumption (CC) begin to infiltrate all facets of our personal lives, encompassing transportation, housing and entertainment, the academic investigation into the adoption intentions for these innovative services remains sporadic and not thoroughly methodical. The academic inquiry into utilising services grounded in CC is a burgeoning and dynamic field. Prior studies have predominantly concentrated on the driving forces and advantages that promote adoption, while the deterrents and potential risks have not been equally examined in academic circles. Additionally, the role of platform attributes has been somewhat neglected. Exploring the barriers to the use of CC services and the significance of platform features is crucial for a couple of reasons: first, the participation in such services may introduce privacy and security concerns for users, potentially undermining the initial motivations; second, the efficacy and success of these services are significantly dependent on the integrity and functionality of the underlying platforms. Thus, this research aims to address the following pivotal question:

*What technical, motivating, and inhibiting factors influence users’ adoption of CC services*?

Drawing on the benefit-risk perspective, this study proposes an integrative theoretical framework that explains the influence of motivating forces (i.e., perceived benefits), inhibiting forces (i.e., perceived risks), and technological forces (i.e., platform quality) on the adoption of CC services. This research is anticipated to provide substantial scientific findings and have practical consequences of great significance. This study enhances the understanding of online CC services by uncovering the relative influence of three independent factors on adoption. This research provides service owners with practical ideas on how to promote the usage of online CC services.

## 2 Literature review

This segment delves into the idea and scholarly investigation of CC. Additionally, it explores the research voids revealed through an examination of existing literature, thereby providing the impetus for our study. This study is only a questionnaire survey on the service perception of car sharing service users and does not involve focus group visits, tests, interviews, etc. Furthermore, this research was conducted by myself as the independent first author, without relying on any unit or organization, and this research has obtained the informed consent of the participants during the questionnaire survey, otherwise the participants can end filling in the questionnaire at any time.

### 2.1 A Working definition of CC

The sharing economy encompasses actions such as sharing, trading and renting products or services without the need of ownership [5]. CC, as first proposed by Felson and Spaeth [6], pertains to situations in which people collectively use economic commodities or services. While the concept of CC isn’t new, its prominence has surged recently thanks to advances in information technology [7]. Modern platforms for CC, like Airbnb and Uber, have played a pivotal role in propelling the sharing economy forward [8]. They achieve this by addressing traditional obstacles to CC, including facilitating user connections, minimizing transaction costs and mitigating risks [9, 10]. Despite the rising interest in the sharing economy and CC, debates over its terminology and definitions persist [11, 12]. This research focuses on examining the factors that motivating, inhibiting, and technologically influence user engagement in CC within an economic model without delving into these terminological debates. Following existing scholarship, this study interchangeably used the phrases sharing economy and CC interchangeably, in line with previous research. These terms refer to the use of technology to facilitate economic models, including sharing, swapping, trading, or renting commodities or services, with monetary or other compensation remuneration [4, 13]. Nevertheless, it does not include non-economic sharing activities, such as those on sites like CouchSurfing.com and BookCrossing.com [12, 14].

### 2.2 Investigation on CC

In an effort to synthesize existing research on CC, a structured review of relevant literature was performed in January 2024. Our literature review process was divided into two phases to ensure a comprehensive identification of pertinent studies. Initially, a search was conducted across prominent databases such as ABI/INFORM Complete, ERIC, ProQuest, and PsycINFO, employing keywords like "CC", "sharing economy", "Uber", and "Airbnb". Additionally, to capture any critical research possibly overlooked, we examined abstracts from articles published in eight leading Information Systems (IS) journals. This initial phase yielded 64 studies for further examination. In the subsequent phase, we refined our pool of studies by applying specific criteria: inclusion was based on whether a study underwent peer-review, and exclusion depended on whether the primary emphasis was on the sharing economy or CC. Following these criteria, 24 studies were deemed relevant for in-depth analysis. Table 1 presents a summary, including the research objectives, theoretical foundation (NIL represents nothing), contexts, methods and analytical levels of these selected studies. Subsequently, we offer a summary of the current state of research on CC, highlighting prevalent trends and patterns observed within the body of literature.

**Table 1:** Summary of prior literature on CC.

Study	Objective	Theoretical foundation	Context	Method	Level
Albinsson and Perera [15]	To explore the drivers and outcomes of participation in CC	Commitment theory	Really Free Markets	Field observation; Interview	I
Ballus-Armet et al., [16]	To analyse the public’s opinion of peer-to-peer automobile sharing and identify prospective market features.	NIL	Peer-to-peer car sharing	Descriptive survey	I
Balnaves [17]	To examine the historical development and distinct characteristics of social media in terms of its role as a platform for mutual help and consumer intimacy.	NIL	Peer-to-peer lending	Case study	M
Bardhi and Eckhardt [18]	To analyse the interactions between consumers and objects, consumers and other consumers, and consumers and marketers in the context of access-based consumption.	NIL	Car sharing	Interview	I
Belk [14]	To evaluate the similarities and distinctions between sharing and CC.	NIL	N/A	Conceptual paper	M
Binninger et al., [19]	To examine the CC paradigm and its impact on sustainable consumption	NIL	46 CC sites & 2 blogs	Content analysis	M
Choi et al. [5]	To present a business model for the sharing economy that is necessary in the introduction and operation among SMEs.	NIL	Tech shop (B2C Tools lending); Shipbuilding Material Joint Distribution Centre (B2B joint purchase)	Case study	M
Cohen and Kietzmann [20]	To investigate developing sustainable business concepts within the shared mobility sector of the sharing economy.	Agency theory	Car sharing	Case study	M
Denning [21]	To analyse the factors that are driving growth of access economy.	NIL	General	Commentary	M
Dillahunt and Malone [1]	To examine the perception and viability of using sharing-economy apps for the purpose of securing temporary work and sharing surplus resources.	NIL	General	Survey Field observation	I
Hamari, Sjöklint, and Ukkonen (In Press) [22]	To examine the reasons behind individuals’ engagement in CC.	Self determination theory	Sharetribe	Survey	I
Henten and Windekilde [23]	To analyse the sharing economy from the standpoint of business modelling and industrial structure.	Transaction cost; Substitution theory	Airbnb; Uber	Case study	M
Martin, Upham, and Budd [24]	To illustrate the commercially-orienting dynamics of a grassroots niche organisations.	Socio-technical transitions theory	Freegle (Online free reuse group)	Case study	M
Matzler, Veider, and Kathan [25]	To identify how companies can react to the rise of sharing economy and adapt their business models.	NIL	General	Commentary	M
McArthur, [26]	To study the motivations to participate in sharing economy activities.	NIL	Landshare (Land sharing)	Content analysis	I
Möhlmann, [27]	To investigate the factors that influence satisfaction and the probability of using a sharing economy alternative.	NIL	Car2go (Car sharing); Airbnb (online community accommodation marketplace)	Survey	I
Nica & Potcovaru, [28]	To examine obstacles and potential advantages for business models that rely on the concept of CC in the fashion sector.	NIL	General	Commentary	M
Pedersen and Netter [29]	To examine the obstacles and potential advantages for business models that are founded on the concept of CC in the fashion sector.	NIL	Fashion library	Case study	M
Zervas et al., [30]	To analyse the influence of Airbnb’s introduction into the Texas market on hotel room income and evaluate the hotels’ market reaction.	NIL	Airbnb	Historical data modelling	M
Benoit et al. [31]	To study multidimensional interpretation of the roles of partcipants in CC.	Capabilities approach	General	Expert survey	I
Boateng et al. [32]	To examine people’ inclination to engage in the sharing economy.	Social exchange theory	Uber	Survey	I
Hong et al. [33]	To study car sharing drivers’ preferences	Latent class logit model	Car sharing	Survey	I
Amat-Lefort et al. [34]	To investigate a novel framework for comprehending the level of excellence in collaborative consuming services.	Parasuraman et al. (1985) GAP model	General	Conceptual paper	M
Amat-Lefort et al. [35]	To examine the viewpoints of both drivers and users on the quality of service provided by sharing economy transport platforms.	NIL	Peer-to-peer transport service	Survey	M

A total of 24 articles investigating CC were published between 2012–2023. The investigation of CC is broad, researchers have examined the phenomenon in different contexts. On one hand, CC was examined in the context of B2C services, such as commercial car sharing [27, 33, 35] and tools lending [5]. On the other hand, CC was explored in the context of C2C services, such as accommodation sharing [30] and task collaboration [1]. Research on CC can be categorised into two levels: analysis at the market level(M) and analysis at the individual level(I), with most research conducted at the market level. There are 14 papers (58%) focusing on market level analysis, while 10 papers (42%) focusing on individual level analysis.

Market-level research on CC is divided into two primary areas of focus. The first group of researchers has developed and examined business models for CC, exploring their applicability across various sectors [19, 34]. For example, through a case study approach, Choi et al. [5] crafted a business model delineating the boundaries and operational strategies for small and medium-sized enterprises (SMEs) aiming to engage in sharing economy ventures. On the other hand, a different set of researchers has delved into understanding the motivators, obstacles, and the effects that CC imposes on conventional businesses [29]. Notably, Henten and Windekilde [23] analyzed the structural changes introduced by the sharing economy through the lens of transaction cost and substitution theories. They argued that the advent of Internet-based platforms for CC significantly lowers transaction costs, thereby serving as a critical catalyst for the expansion of sharing economy practices. In a similar vein, Zervas et al. [30] scrutinized the impact of Airbnb’s entry on hotel revenues in Texas, employing historical data analysis. They found that every 10% rise in Airbnb listings led to a 0.35% fall in monthly hotel room revenues, with the effect being most pronounced in Austin, where Airbnb’s presence is strongest, culminating in a revenue impact surpassing 13%.

The research on individual-level analysis remains relatively limited in the literature. Existing studies mainly explored the motivational factors that predict users’ participation in CC activities. Extrinsic motivations (such as economic gain) [22] and satisfaction [27]) and intrinsic motivations (such as belonging [26] and trust [27]) were both found important in influencing users’ participation in CC. For instance, Ballus-Armet et al. [16] and Amat-Lefort et al. [35] conducted an intercept survey regarding public perception of peer-to-peer car sharing, and concluded that convenience and availability, monetary savings, trust and expanded mobility options were essential motivators that entice users to use the car sharing services.

In sum, the research on CC is still in its developmental stage as reflected by two research gaps in the review of the literature. First, existing studies are mainly conceptual and framework papers. Factors affecting users’ participation in CC activities are rarely validated empirically. Second, there is scant knowledge regarding the inhibiting factors and technological factors associated with CC. Since the Internet has extended the CC activities beyond a small network of known members, individuals nowadays can now interact with people around the world in the CC platforms. Such dependence on the platform and uncertainty involved in interacting with complete strangers signify the need to understand (1) the risk factors that deter users’ participation and (2) technological factors that facilitate users’ participation in CC activities.

## 3 Hypothesis established

### 3.1 Model conceptualization

**Fig 1:**
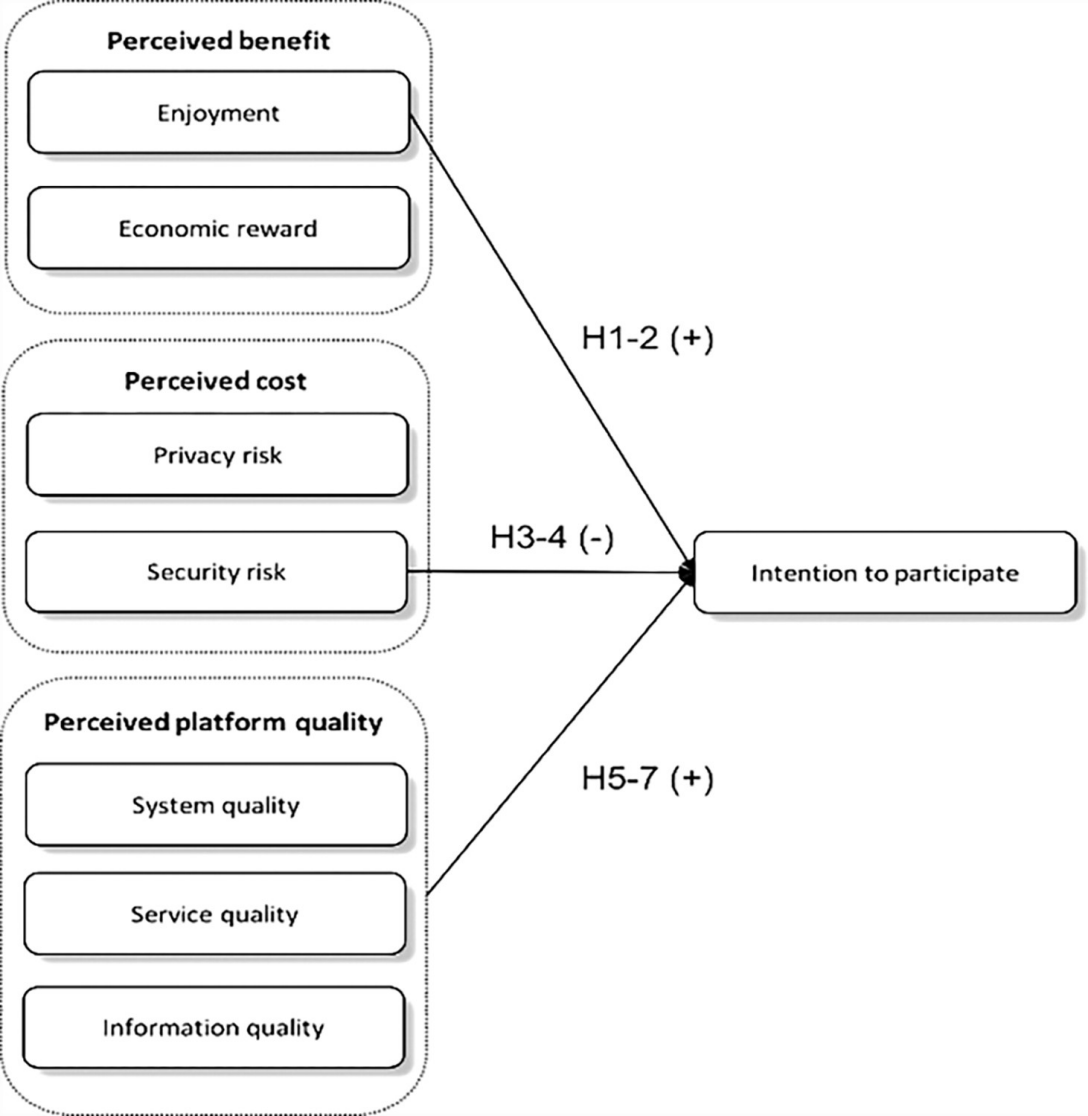
Proposed research model.

The technology acceptance model is commonly used to study the uses of information systems and technologies. However, the cost and benefit framework is widely adopted when studying the uses of IS/IT that involve potential risks, such as privacy and security risks. Examples of studies that have used this framework include Krasnova, Spiekermann, Koroleva, & Hildebrand [36] and Zhou, Lu, & Wang [37]. The previous research extensively documents the possible advantages of engaging in CC, including economic incentives, pleasure, sustainability, reputation and perceived service quality [22, 26, 35]. Nevertheless, there is a rising apprehension over the possible hazards associated with engaging in CC, which have been largely disregarded in previous studies. Hence, this study utilises the cost and benefits framework and presents a research model to comprehensively investigate the impact of perceived costs and perceived advantages on user intention to engage in CC. Furthermore, the study model investigates platform quality’s significance in modern sharing economies, where collaborative consuming behaviours are facilitated and enhanced by sophisticated electronic platforms. Fig 1 illustrates the research model.

### 3.2 Identified perceived benefits

The literature previously identifies two primary categories of perceived benefits linked with engaging in CC: enjoyment and reputation, which are considered intrinsic rewards, along with economic benefits and environmental sustainability, categorized as extrinsic rewards [22]. Although this research is focused on exploring the determinants that affect individual participation in CC from the standpoint of users/consumers, it does not cover antecedent factors related to the organizational or societal level, particularly those associated with the provider like reputation and environmental sustainability.

#### 3.2.1 Intrinsic benefit: Enjoyment

In this context, enjoyment signifies how the act of participating in CC is intrinsically pleasurable, independent of any expected performance outcomes [38]. Enjoyment is acknowledged as a key intrinsic advantage associated with online sharing activities across diverse technological environments [22]. For example, Nov [39] discovered that enjoyment emerged as the most prominent motivator for contributing to Wikipedia, outshining other types of intrinsic motivation. Similarly, Roberts, Hann, and Slaughter [40] observed that software developers find pleasure in contributing to open source software projects.

Likewise, enjoyment is anticipated to play a crucial role in encouraging participation in CC within the context of the sharing economy. Specifically, McArthur [26] and Li & Shang [41] posited that engaging with platforms for sharing accommodation (e.g., Airbnb) captivates users by offering them a unique and genuine experience. Consequently, it is proposed that:

*H1*: *The intention of users to engage in collaborative consuming is positively correlated with their perceived level of Enjoyment*.

#### 3.2.2 Extrinsic benefit: Economic reward

Users motivated intrinsically engage with information systems/technology (IS/IT) for the enjoyment gained from such interactions, whereas those with extrinsic motivation seek external rewards or benefits, such as economic incentives [42]. An essential extrinsic motivator for participating in CC is the economic benefit it offers. The appeal of the sharing economy to numerous consumers stems from its cost-effectiveness [23]. Fundamentally, the sharing economy and CC are built on the idea of sharing assets among individuals rather than owning them outright [16]. As such, CC is often viewed as behavior aimed at maximizing utility, where users opt for more affordable alternatives to owning goods and services.

This maximization of utility is facilitated by providing temporary, non-ownership access to idle personal assets [28]. Additionally, sophisticated digital platforms bridge the gap between providers and users of these goods and services, allowing for CC on a larger scale [28]. As a result, the goods and services on modern CC platforms are offered at reduced prices, bolstering participation. The link between economic benefits and the intention of users to engage in CC has been affirmed in earlier studies [22, 43]. It is, therefore, proposed that:

*H2*: *The intention of users to engage in collaborative consuming is positively correlated with their perceived level of economic reward*.

### 3.3 Identified perceived costs

CC has been linked to a variety of advantages, including enjoyment, economic reward, improved company sustainability, and lower environmental pollution [22, 27]. Participating in CC, however, is not without risks. Indeed, growing concerns on potential risks of such activities have been raised [44]. Specifically, recent studies revealed that privacy and safety are the major concerns of participating in CC activities [1].

#### 3.3.1 Perceived privacy risk

In this research, privacy risk is defined as the potential for improper gathering and utilization of personal user data by providers of CC platforms [45]. The necessity to submit considerable amounts of personal information on these platforms constitutes a significant worry for users [16], deterring them from engaging in CC activities [1].

Certain online corporations are known to exploit users’ personal data opportunistically to achieve extra financial benefits, leading to privacy concerns becoming a predominant worry for users [46]. Privacy risk, in particular, is recognized as a significant deterrent across various online activities. For example, individuals often engage in a risk-benefit assessment when asked to share personal data with organizations [47]. Moreover, services online that are personalized and location-based demand more sensitive information, such as users’ demographics, usage history, and location details, thereby elevating the privacy risk levels [48]. The adverse association between privacy risks and online engagement has been established in existing research [49, 50]. In a similar vein, CC platforms necessitate the submission of comprehensive personal information by users, including demographic details, social connections, digital activity logs, and location data, which adversely affects their readiness to engage in sharing economy ventures [1]. Based on these insights, the hypothesis is proposed that:

*H3*: *The intention of users to engage in collaborative consuming is negatively correlated with their perceived level of privacy risk*.

#### 3.3.2 Perceived security risk

In this research, security risk is defined as the potential for adverse outcomes affecting individuals or network resources due to specific conditions, situations, or events [51]. Within the realm of CC, security risks may emerge from damage to personal belongings and assets or result in physical harm to the users. Such risks have been identified as significant deterrents to the use of various online platforms, including e-commerce [52], social media sites [53], and mobile banking services [54].

Security concerns are a prevalent issue in the modern practice of CC. For example, incidents of assault, property damage, and theft have been reported by users of accommodation sharing services, notably on the well-known platform Airbnb [55]. Additionally, participants in NeighborGoods, a digital community for the local exchange of goods, expressed a higher likelihood of engaging in sharing activities if secure exchange locations, such as police or fire stations, were facilitated by the platform [1]. Concerns over insurance coverage and liability have been raised by users of peer-to-peer car sharing services, highlighting worries about the safety of their vehicles during the exchange [16]. As users of platforms like Airbnb and Uber actively partake in sharing activities, the threat to security poses another significant hurdle, potentially discouraging their participation in such economic interactions. Based on these observations, the following hypothesis is proposed:

*H4*: *The intention of users to engage in collaborative consuming is negatively correlated with their perceived level of security risk*.

### 3.4 Identified perceived platform quality

Platform Quality refers to the service quality content and functions provided via a CC platform in fulfilling users’ goals, which includes three parts of quality assessment, service quality, system quality and information quality [41, 56, 57]. Service quality, System quality and information quality directly shape users’ attitudes towards satisfaction [58]. Based on communication theory, Gorla et al. [59] believe that information is the result or outcome of several systems, including data processing, communication medium, and entertainment systems. Given that information is generated by a system, any issues with the system’s quality might diminish the overall quality of the information it generates, thus reduce the user’s overall service quality perceptions. Therefore, these three platform qualities should be combined instead of only focusing on one of them [27, 35].

#### 3.4.1 Perceived system quality

Perceived system quality is the user’s evaluation of the system’s technical capabilities and usability. Research in recent years has shifted the focus to decomposing the evaluation of CC service quality from offline into online [41, 60]. Researchers have attempted to identify key online service attributes to reduce inaccurate methods for assessing the service quality of this nascent online platform [61] and studied the joint impact of perceptions of platform online system quality on continuous use intention [60].

Measuring the system quality for CC services can improve the operational efficiency of the platform and guide the platform to better service users during peak use time and congested periods [62]. What is more, it can bring a good sense of user experience to users and, at the same time, improve the economic income and service quality of the platform, hence increase the user intention to participate in CC [41]. Therefore, it is hypothesized that:

*H5*: *The intention of users to engage in collaborative consuming is positively correlated with their perceived level of System quality*.

#### 3.4.2 Perceived service quality

Perceived Service quality refers to the overall platform quality perception obtained during the use in CC [63]. Service quality is the achievement of user service [64]. Users will form service expectations from past experience, word of mouth and marketing communication. In each service encounter, users will compare the actual perceived service with the service expectation [65]. In a word, if the perceived service quality is not as good as the service expectation, users will be disappointed.

Service quality has been recognised as a key driver to attract and retain users in the service literature [66]. Prior research has indicated that Service quality is a strong antecedent of user satisfaction [67] and loyalty [68], and that user satisfaction and loyalty are important indicators to measure the success of a company. Therefore, it is hypothesized that:

*H6*: *The intention of users to engage in collaborative consuming is positively correlated with their perceived level of Service quality*

#### 3.4.3 Perceived information quality

In contrast, perceived information quality is the user’s evaluation of the system’s semantic communication and/or knowledge exchange. For CC, the information system’s output is through CC platform. According to DeLone & McLean [69], information quality may be broken down into three categories: accuracy, completeness and currency. Accuracy means the extent to which users perceive the information from the CC platform is accurate while completeness means the extent to which users perceive the information from the CC platform is complete [60]. Unlike accuracy and completeness, currency means the extent to which users perceive the information from the CC platform is up to date [62].

Based on communication theory, information quality of the platform in CC is paramount because it underpins trust and decision-making among participants [59]. High-quality information facilitates transparency, enabling users to make informed choices based on accurate, complete, and timely data [27]. It enhances user satisfaction, fosters trust in the platform, and reduces perceived risk associated with transactions involving goods or services shared between individuals [70, 71]. Consequently, information quality directly impacts the platform’s reputation, user retention, and the overall success of the CC ecosystem. Therefore, it is hypothesized that:

*H7*: *The intention of users to engage in collaborative consuming is positively correlated with their perceived level of Information quality*

## 4 Methodology of the research

### 4.1 Sample and context of the research

This study validated the suggested research model by conducting tests on 524 active customers of car-sharing services in Hong Kong. One of the five most important parts of the sharing economy, according to a new survey by PwC, is automobile sharing/car sharing. Take Uber as an example, this five-year-old car-sharing company has grown to serve more than 250 cities across four continents [3]. CC has started to take off in Hong Kong [72]. It is found that about 30% of the Internet users has engaged in CC activities in the past year, while car sharing ranked the top among all CC activities. Characterized by the prevalence of CC activities and salient participation of car sharing activities in Hong Kong, it is thus, appropriate to select users in Hong Kong as the current research context and sample.

### 4.2 Items generation

The measurement items have been drawn from previous studies, with some adjustments to suit the contemporary context of CC. Several measuring questions were created for each construct and assessed using a seven-point Likert scale, ranging from "1 = Strongly Disagree" to "7 = Strongly Agree". Table 2 displays the measurement items.

**Table 2:** Measurement items.

Construct	Items	Reference
Enjoyment	I find participating in that CC activity enjoyable.	Venkatesh and Morris [73]
	Participating in that CC activity is pleasant.	
	I have fun in participating in CC activity.	
Economic reward	Engaging in that CC activity is more cost-effective compared to other possibilities in the market.	Kim, Chan, and Gupta [74]
	I am able to accumulate more savings by engaging in that joint consumption activity.	
	Participating in such collaborative consumer activity may result in a more favourable discount.	
Privacy risk	Engaging in such collective consumer behaviour poses privacy hazards.	Malhotra, Kim, and Agarwal [75]
	Engaging in such CC activity carries the risk of possible privacy infringement.	
	There are several unforeseen privacy concerns that arise while engaging in CC activities.	
Security risk	Engaging in that CC activity would be insecure.	Grewal, Munger, Iyer, and Levy [76]
	Participating in that CC activity is not safe.	
	Participating in that CC activity is insecure.	
Service Quality	The CC platform responds quickly to my inquiries.	Jayawardhena [77]
	I may contact a representative at the CC platform if I have any issues with my account.	
	When I enter my account, I feel safe; the CC platform gives me confidence.	
	The CC platform recognises its consumers’ demands.	
	The CC platform offers the service precisely as advertised.	
System Quality	The CC platform allows me to get to it fast.	Wixom and Todd [58]
	Every time, the CC platform works as promised.	
	The CC platform is flexible enough to accommodate many requirements.	
	I think the CC platform is an outstanding system.	
	Accessing any part of the site is a simple using the CC platform.	
Information Quality	I may get the latest data from the CC platform.	Wixom and Todd [58]
	Everything I need is supplied to me via the CC platform.	
	According to the joint consumption platform, the data is correct.	
	As a whole, the data that I get from the CC platform is really good quality.	
Intention to participate in CC	I want to engage in CC at some point in the future.	Ajzen [78]
	I plan to engage in collaborative consuming to meet my future demands.	
	In the future, I see myself engaging in CC.	

### 4.3 Pre-test

A pre-test was performed involving 30 participants who had previous experience with car-sharing services in December 2023, aiming to gather insights on the questionnaire design. Participants provided feedback regarding the clarity of the survey instructions, its overall flow and the phrasing of the questions. While only minor adjustments were made to the survey’s format, the assessment revealed no significant issues.

### 4.4 Gathering data and analysis

For our comprehensive field study, we utilized an online questionnaire in January 2024 as the primary method for data collection to evaluate our theoretical framework. This choice was predicated on the assumption that individuals with a background in CC would possess advanced Internet skills. The survey initiated with a preliminary query to ascertain respondents’ experience with car-sharing services. Following this, an introduction outlining the study’s objectives and a consent agreement was provided. Participants consenting were then navigated through the main body of the questionnaire, which included reflections on their latest car-sharing experience. It probed into their views on participating in CC, factors that encourage or discourage participation, and the role of technological influences. Additionally, it gathered demographic details such as gender, age, income, educational background, and frequency of Internet use. To ensure a diverse and representative sample of the CC community, participants were sourced through a marketing research agency.

The analysis of our theoretical model was conducted using Structural Equation Modeling (SEM) methods. SEM is recognized for its precision and adaptability in discerning the dynamics between various elements and outcome variables, while also accounting for measurement inaccuracies [79]. This method is deemed essential for the analysis in our study, providing a clean and accurate model evaluation.

#### 4.4.1 Informed consent

This study strictly abides by the ethical regulations of Northumbria University, respects the personal wishes of the participants when conducting interviews, and signs content forms. Because this study is a pure quantitative method, online questionnaires were delivered to respondents. Prior to dissemination, respondents will be notified of the study’s topic as well as its substance. Prior to starting the research, their permission will be asked. Respondents should not be allowed to learn the identity of study participants. Respondents have the option to opt out of the survey at any time. Data acquired for study must be utilised only for this purpose. To ensure that the acquired data is not modified, the data collected for this project cannot be shared with outsiders. This research was assessed for the importance of research ethics and complies with the University (Northumbria) Policy Statement on Research Ethics, which will ensure the confidentiality and anonymity of respondents.

### 4.5 Model validating

The model validating of this empirical study was implemented through SPSS26 and AMOS24. Regarding research methods, data characteristics that satisfy normal distribution are described in the form of mean and standard deviation. For the content reliability of the scale, the Cronbach’s alpha reliability test was used to assess the internal consistency of the scale. The validity test uses the CFA paradigm for confirmatory factor analysis to assess construct validity, convergent validity, combined reliability and discriminant validity. Finally, the correlation and influence relationship between variables were tested using Pearson correlation analysis and structural equation model (SEM) respectively.

#### 4.5.1 Description of the distribution of basic demographic characteristics of the survey target group

Table 3 presents the demographic characteristics of the respondents (n = 524). The gender distribution is relatively balanced, with 256 males accounting for 48.9% and 268 females accounting for 51.1%. The majority of respondents were aged under 45 years old, among them there are 195 aged 36–45 years old, accounting for 37.2% of the sample. Overall, the sample group is younger. Regarding education level, except for the small proportion of sample groups with master’s degrees at 4.4%, the overall academic level is relatively balanced, ranging from 20% to 26%. In terms of monthly income level, the majority of respondents earned between HK$5,000 and 16,000, accounting for 48.1% of the sample.

**Table 3:** Respondent profile.

Variable	Options	Frequencies	Percent
Most Car sharing platforms used	Uber	302	57.6%
	Lyft	70	13.4%
	Ola	58	11.1%
	Didi	64	12.2%
	Other	30	5.7%
Age	18–25	166	31.7%
	26–35	140	26.7%
	36–45	195	37.2%
	46–55	8	1.5%
	55–65	8	1.5%
	66	7	1.3%
Car sharing frequency/Internet-usage	Never	15	2.9%
	Occasionally	22	4.2%
	Sometimes	32	6.1%
	Usually	242	46.2%
	Always	213	40.6%
Gender	Male	256	48.9%
	Female	268	51.1%
Educational background	Less than high school	127	24.2%
	High school	123	23.5%
	College	119	22.7%
	Bachelor	132	25.2%
	Master or above	23	4.4%
Monthly income	No income	14	2.7%
	Less than 5000	115	21.9%
	5000–8999	127	24.2%
	9000–15999	125	23.9%
	16000–23999	78	14.9%
	24000–31999	25	4.8%
	32000–39999	21	4.0%
	40000 or more	19	3.6%
Number of car sharing /past week	1	49	9.4%
	2	64	12.2%
	3	92	17.6%
	4	127	24.2%
	5	124	23.7%
	6	43	8.2%
	7 or more	25	4.8%
Experience	Less than 1 year	27	5.2%
	1–2 years	34	6.5%
	3–4 years	136	26.0%
	5–6 years	138	26.3%
	7–8 years	147	28.1%
	9–10 years	42	8.0%

Uber was the most frequently used car sharing service in the prior CC experience, accounting for 57.6% of respondents. The majority of respondents reported using Car-hailing services at the " usually " level, accounting for 46.2%. The most frequently used frequency of car sharing services in the past week was four times, accounting for 24.2%. The majority of Car-hailing software users had 7–8 years of experience (28.1%), followed by 5–6 years (26.3%).

#### 4.5.2 Reliability test

The data of perceived benefits, perceived costs, perceived platform quality and intention to participate in CC involved in this study were all collected using scales. In this analysis, Cronbach’s α was used to conduct a reliability test (Table 4). In the actual measurement results, the perceived benefits were 0.835, the perceived costs were 0.82, the perceived platform quality was 0.896, and the willingness to participate was 0.863. The measured reliability coefficient results are all above 0.8, indicating that the scales used in this study have good internal consistency and reliability.

**Table 4:** Reliability test.

Factors	Cronbach’s α	Items
Enjoyment	0.852	3
Economic reward	0.820	3
**Perceived Benefits**	**0.835**	**6**
Privacy risk	0.917	3
Security risk	0.914	3
**Perceived Costs**	**0.824**	**6**
System Quality	0.883	5
Service Quality	0.871	5
Information Quality	0.856	4
**Perceived Platform Quality**	**0.896**	**14**
Intention to participate in CC	**0.863**	**3**

#### 4.5.3 Confirmatory factor analysis

The confirmatory factor analysis (CFA) findings indicate that the model fitness test results were as follows: CMIN/DF (chi-square degree of freedom ratio) = 2.091, RMSEA (root mean square error) = 0.046, NFI (normed fit index) = 0.926, RFI (relative fit index) = 0.914, IFI (incremental fit index) = 0.960, TLI (Tucker-Lewis index) = 0.953, and CFI (comparative fit index) = 0.96. The measured values of each fitting indicator fall within the range of excellence, suggesting that the CFA model demonstrates a satisfactory level of fit. Moreover, the suggested scale has strong structural validity.

In the convergent validity and combined reliability tests (Table 5), the factor loadings of each measurement item were all above 0.7, indicating that each measurement item has a strong ability to explain each latent variable. Among them, Enjoyment (ENG)’s AVE = 0.655, CR = 0.851; Economic reward (ECO)’s AVE = 0.601, CR = 0.817; Privacy risk (PRI)’s AVE = 0.787, CR = 0.917; Security risk (SEC)’s AVE = 0.781, CR = 0.915; System Quality (SYS)’s AVE = 0.601, CR = 0.883; Service Quality (SER)’s AVE = 0.577, CR = 0.872; Information Quality (INF)’s AVE = 0.594, CR = 0.854 and Intention to participate in CC (INT)’s AVE = 0.677, CR = 0.862. The findings of the convergent validity and combined reliability analysis for each component indicate that all factors have strong convergent validity and combined reliability, with values above 0.5 and 0.7, respectively.

**Table 5:** Convergent validity.

Path relationship			Estimate	AVE	CR
ENG3	<—	ENG	0.835	0.655	0.851
ENG2	<—		0.836		
ENG1	<—		0.755		
ECO3	<—	ECO	0.784	0.601	0.817
ECO2	<—		0.807		
ECO1	<—		0.733		
PRI3	<—	PRI	0.880	0.787	0.917
PRI2	<—		0.911		
PRI1	<—		0.870		
SEC3	<—	SEC	0.873	0.781	0.915
SEC2	<—		0.887		
SEC1	<—		0.891		
SYS5	<—	SYS	0.780	0.601	0.883
SYS4	<—		0.814		
SYS3	<—		0.785		
SYS2	<—		0.771		
SYS1	<—		0.724		
SER5	<—	SER	0.768	0.577	0.872
SER4	<—		0.757		
SER3	<—		0.735		
SER2	<—		0.788		
SER1	<—		0.750		
INF4	<—	INF	0.764	0.594	0.854
INF3	<—		0.809		
INF2	<—		0.812		
INF1	<—		0.693		
INT3	<—	INT	0.845	0.677	0.862
INT2	<—		0.860		
INT1	<—		0.759		

Note: ENG is Enjoyment; ECO is Economic reward; PRI is Privacy risk; SEC is Security risk; SYS is System Quality; SER is Service Quality; INF is Information Quality; INT is Intention to participate.

The discriminant validity test (Table 6) involves comparing the correlation coefficient between components with the square root of the average variance extracted (AVE). The correlation coefficient between each element in the actual measurement findings ranges from 0.2 to 0.75, whereas the square root of AVE ranges from 0.77 to 0.89. By comparing the correlation coefficients between each component, it becomes evident that they are all lower than the square root of AVE. This indicates that each factor of the scale has strong discriminant validity.

**Table 6:** Discriminant validity.

Factors	ENG	ECO	PRI	SEC	SYS	SER	INF	INT
ENG	**0.655**							
ECO	0.559	**0.601**						
PRI	-0.243	-0.284	**0.787**					
SEC	-0.407	-0.367	0.265	**0.781**				
SYS	0.556	0.554	-0.315	-0.366	**0.601**			
SER	0.600	0.492	-0.339	-0.341	0.524	**0.577**		
INF	0.534	0.570	-0.332	-0.339	0.484	0.524	**0.594**	
INT	0.701	0.660	-0.365	-0.464	0.745	0.689	0.724	**0.677**
**SQRT(AVE)**	**0.809**	**0.775**	**0.887**	**0.884**	**0.775**	**0.760**	**0.771**	**0.823**

Note: ENG is Enjoyment; ECO is Economic reward; PRI is Privacy risk; SEC is Security risk; SYS is System Quality; SER is Service Quality; INF is Information Quality; INT is Intention to participate.

According to the findings of the reliability and validity tests in this section, the scale used in this research demonstrates strong reliability and validity.

#### 4.5.4 Variable descriptive statistics

Through descriptive statistical analysis (Table 7), perceived benefits (5.281±1.371), including two factors: enjoyment (5.317±1.54) and economic reward (5.246±1.533).

**Table 7:** Variable descriptive statistics (N = 524).

Variables	Minimum	Maximum	Average	Deviation
**Perceived Benefits**	1	7	5.281	1.371
Enjoyment	1	7	5.317	1.540
Economic reward	1	7	5.246	1.533
**Perceived Costs**	1	7	4.606	1.497
Privacy risk	1	7	4.644	1.862
Security risk	1	7	4.568	1.847
**Perceived Platform Quality**	1	7	5.236	1.318
System Quality	1	7	5.034	1.344
Service Quality	1	7	5.337	1.107
Information Quality	1	7	5.491	1.635
**Intention to participate**	1	7	5.300	1.518

Perceived cost: Perceived cost (4.606±1.497), including privacy risk (4.644±1.862) and security risk (4.568±1.847).

Perceived system security: Perceived system security (5.236±1.318), including system quality (5.034±1.344), service quality (5.337±1.107) and information quality (5.491±1.635).

Intention to participate: The mean and standard deviation of willingness to participate are 5.3±1.518.

The scale is a 7-point Likert scale, and the scale score is calculated by calculating the mean, so 4 is the theoretical medium level. It can be seen from the actual measurement results that the mean level of each factor is above 4. Overall, the respondent group has a high level of agreement on each measurement factor. Among them, the mean value of perceived cost is relatively low because the level of perceived risk is low.

#### 4.5.5 Testing the correlation between variables

The Pearson correlation analysis was used to examine the correlation between variables (Table 8). The test findings indicate a notable association across many factors, particularly a substantial negative correlation between Privacy risk, Security risk, and Intention to participate. Furthermore, there exists a substantial positive link between Enjoyment, Economic reward, Service quality, System quality, Information quality, and Intention to engage. The magnitudes of the correlation coefficients range from 0.2 to 0.75, indicating a moderate to weak association between the variables.

**Table 8:** The correlation between variables.

Variables	Intention participate	Enjoyment	Economic reward	Privacy risk	Security risk	System Quality	Service Quality	Information Quality
Intention participate	1							
Enjoyment	.593**	1						
Economic reward	.546**	.452**	1					
Privacy risk	-.322**	-.206**	-.242**	1				
Security risk	-.409**	-.353**	-.313**	.244**	1			
System Quality	.641**	.466**	.459**	-.282**	-.326**	1		
Service Quality	.600**	.511**	.410**	-.304**	-.304**	.459**	1	
Information Quality	.604**	.440**	.462**	-.285**	-.289**	.401**	.441**	1

** Correlation is significant at the 0.01 level (two-tailed).

#### 4.5.6 Structural equation model

**Fig 2:**
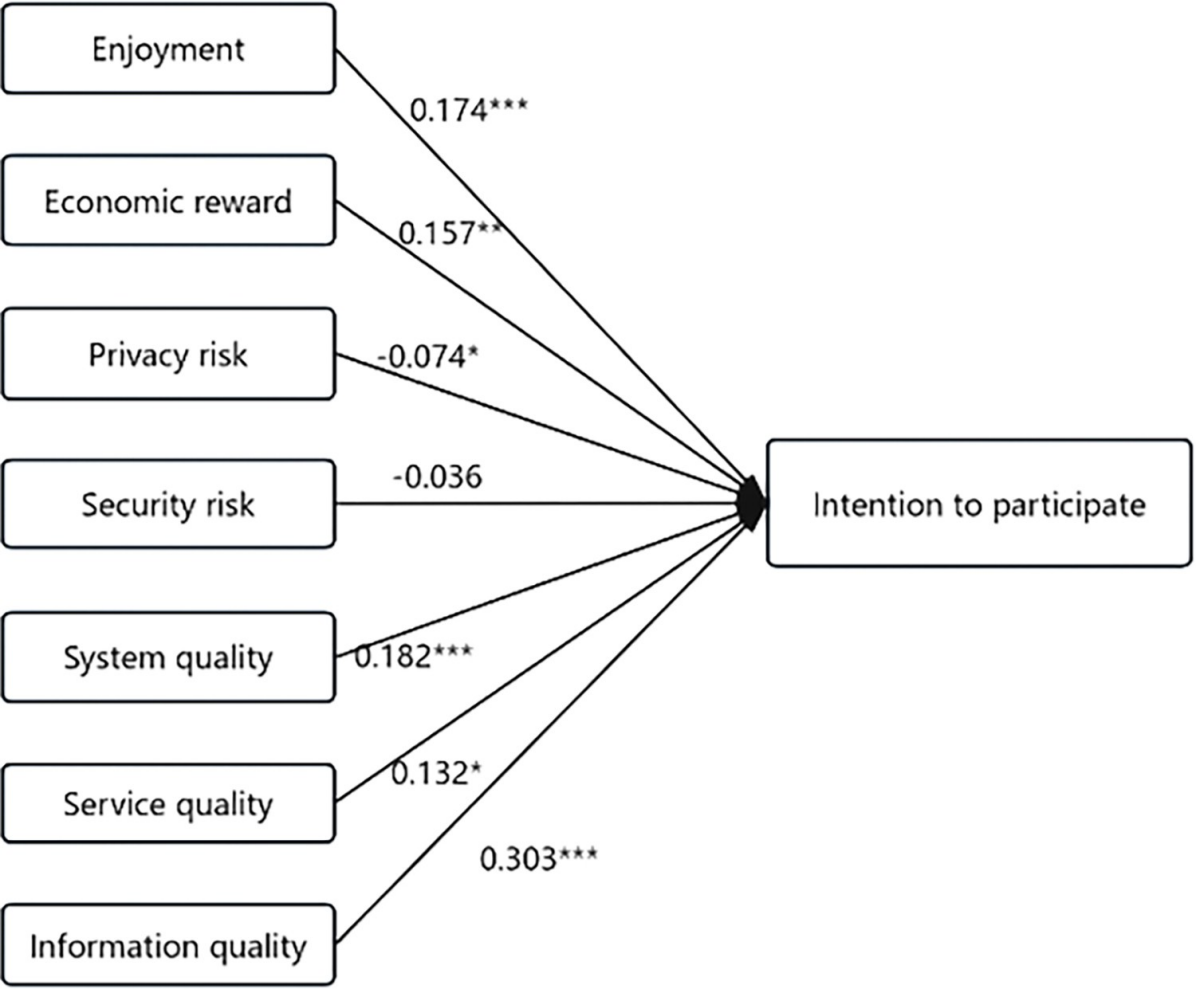
SEM model diagram.

*4*.*5*.*6*.*1*. *SEM model fitness test*. Construct an SEM model based on the primary hypothesis relationship to test the influence relationship between variables (Fig 2). In this test, the fitness test results of the constructed structural equation model were: CMIN/DF = 2.091, RMSEA = 0.046, NFI = 0.926, RFI = 0.914, IFI = 0.96, TLI = 0.953 and CFI = 0.96. The measured results of each fitting index are all within the excellent level, indicating that the SEM model constructed in this study has good fitness.

*4*.*5*.*6*.*2*. *SEM model path relationship test*. The factor test results in Table 9 indicate that for Intention to participate, Enjoyment has a significant positive impact (β = 0.158, p<0.001). Economic reward also has a significant positive impact (β = 0.097, p<0.05), while Privacy risk does not have a significant impact (β = -0.013, p>0.05). On the other hand, Security risk has a significant negative impact (β = -0.075, p<0.05). System quality (β = 0.33, p<0.001), Service quality (β = 0.188, p<0.001), and Information quality (β = 0.296, p<0.001) all have significant positive relationships.

**Table 9:** SEM model path relationship test.

Path relationship			Estimate	S.E.	C.R.	P
INT	<—	ENG	0.158	0.042	3.384	***
INT	<—	ECO	0.097	0.045	2.130	0.033
INT	<—	PRI	-0.013	0.025	-0.424	0.671
INT	<—	SEC	-0.075	0.025	-2.268	0.023
INT	<—	SYS	0.330	0.044	7.428	***
INT	<—	SER	0.188	0.045	4.285	***
INT	<—	INF	0.296	0.047	6.571	***

Note: *** is significant at the 0.001 level (ENG is "Enjoyment", ECO is "Economic reward", PRI is "Privacy risk", SEC is "Security risk", SYS is "System quality", SER is "Service quality" ", INF is "Information quality")

*4*.*5*.*6*.*3*. *Summary of hypothesis testing results*. Hypothesis testing was conducted through SEM model, in which the test result of privacy risk was not supported. The test results of other factors are supported.

## 5. Discussion

This study aims to deepen our understanding of the motivating and inhibiting factors that influence users’ participation in collaborative consumption. Based on the cost and benefit framework, this paper proposes a model to explore the influences of perceived benefits (enjoyment and economic reward), perceived costs (privacy risk and security risk) and perceived platform quality (system quality, service quality and information quality). Based on our analysis, more than 50% of participants are willing to participate in CC (Table 7), which is consistent with the view held by theoretical research that the sharing economy will become the future mainstream consumption model. In addition, this study has two notable findings. First, in the context of CC-driven car sharing business, four cost-benefit factors of intention to participate and three quality factors under the platform dimension are proposed and verified. The study found that perceived enjoyment and economic reward, perceived security risk and privacy risk, perceived system quality, service quality and information quality are all crucial factors that influence users’ intention to participate in CC. In this study, by analysing questionnaire data, the test factors are indicators of the perceived benefit dimension, perceived cost dimension and perceived platform quality dimension.

Secondly, we verified the relationship between perceived benefits, perceived costs and intention to participate CC from the perspective of costs and benefits of car sharing services driven by CC. The business we are studying is conducted through Internet technology—the online platform for CC. Therefore, we also verified the impact of the quality aspects of CC platforms on the intention to participate. Most of these relationships are supported in our research context. It can be seen from Table 10 that perceived benefits, perceived platform quality and the five factors below all positively affect users’ intention to participate in CC. However, the perceived cost shows a relationship between partial support and intention to participate in CC, in which the privacy risks we usually think of are not supported, but the security risks are supported. This is consistent with previous research that indicates that the security factor has always been regarded as an important influencing factor for users to participate in the collaborative economy [27]. It is also one of the critical dimensions for evaluating the CC services quality [80, 81].

**Table 10:** Summary of hypothesis testing results.

Hypothesis	Supported
Perceived benefits have a significant positive impact on intention to participate	Yes
H1	Yes
H2	Yes
Perceived cost has a significant negative impact on intention to participate	Part
H3	No
H4	Yes
Perceived platform quality has a significant positive impact on intention to participate	Yes
H5	Yes
H6	Yes
H7	Yes

## 6. Contributions

This study enhances the knowledge in the sharing economy domain by identifying the factors that influence individuals’ involvement in CC. This study presents a model that outlines the elements that influence user involvement in CC. The model is built on a theoretical framework that uses cost and benefit analysis to examine the motivational, deterrent, and technical aspects. The validity of the model was verified by analysing survey data and using structural equation modelling. This inquiry provides substantial theoretical and practical knowledge in the field of collaborative consuming. Theoretically, it broadens the discussion on information systems (IS) by examining digitally enabled CC, which has not been extensively studied. This study investigates the deterrent and technical aspects associated with CC, while also confirming the incentive variables identified in previous studies. The existing study paradigm provides a strong foundation for future investigations on digitally facilitated CC. Essentially, the results provide industry experts with recommendations on the specific aspects at the individual level that affect CC. An analysis of technical factors provides important suggestions for improving the quality of the platform and developing tactics to decrease the perceived dangers among users.

## 7. Limitations and future research directions

The study also has several limitations that provide opportunities for future research. First, while the study attempted to cover benefits and cost critical dimensions of in CC, some dimensions considered critical by car sharing users were left out of the analysis. Future research could explore the inclusion of other factors and examine their importance in evaluating users participate intention.

Second, this study actually develops a scale that can be used by CC companies to assess aspects of user care, but this study does not examine the nomological validity of the new scale in a model of continuing use intention for information systems. Future research could explore the use of the new scale in other research areas beyond the continuing use intention of information systems.

Third, the study collected data from car sharing users in Hong Kong, and the findings may not generalize to other cultures and regions. Additionally, the study did not include other largest car sharing services popular in other areas or countries, such as Didi travelling. Future research could replicate the study in different cultural contexts and include other sharing economy users to test and extend the generalizability of the scale.

In conclusion, the study provides a foundation for future research to address these limitations and explore the dimensions and factors that affect CC users’ intention to participate from different perspectives and in other contexts.
